# Flexor Digitorum Profundus Tendon Avulsion With Large Extra-articular Bony Fragment Secondary to Dog Collar Entrapment: A Case Report

**DOI:** 10.7759/cureus.91293

**Published:** 2025-08-30

**Authors:** Armandeep Bedi, Prashanth D'sa, Madan K Sathyanarayanan, Rahul Kotwal

**Affiliations:** 1 Trauma and Orthopaedics, NHS Wales, Cardiff, GBR; 2 Trauma and Orthopaedics, University Hospital Sussex NHS Foundation Trust, Brighton, GBR; 3 Cardiothoracic Surgery, Morriston Hospital, Swansea, GBR; 4 Trauma and Orthopaedics, Princess of Wales Hospital, Bridgend, GBR

**Keywords:** fdp injury, flexor tendon rupture, jersey finger, low-trauma fractures, unusual injury

## Abstract

Jersey finger (or rugby finger) refers to an avulsion of the flexor digitorum profundus (FDP) tendon, where it is torn from its distal attachment on the distal phalanx. FDP avulsion injuries typically occur in contact sports due to forced extension of the distal interphalangeal joint (DIPJ) while the finger is actively flexing. We present a unique case involving a 56-year-old female patient who sustained a rare pattern of FDP avulsion injury while attempting to restrain her dog via its collar. The patient presented to the accident and emergency (A&E) 12 hours following the incident. Radiographs revealed an unusually large extra-articular bony fragment of the distal phalanx (approximately 80% of its length) displaced volarly, resting against the middle phalanx. The patient was evaluated by both orthopaedics and plastic surgery. The injury was managed through open reduction and fixation with a Kirschner wire, resulting in a complete functional recovery following 2-3 months of regular physiotherapy sessions post-operatively. This injury pattern is underrepresented in the literature, as it involves an extra-articular disruption of the flexor digitorum, a mechanism that has not been described before. Early recognition and surgical intervention are critical for optimal outcomes.

## Introduction

The term 'jersey finger' refers to an avulsion of the flexor digitorum profundus (FDP) tendon at its distal phalangeal insertion, typically occurring when an athlete forcibly grabs an opponent’s jersey, leading to sudden hyperextension of the distal interphalangeal (DIP) joint during active flexion [1]. While commonly associated with sports, non-sport-related mechanisms have also been documented, including injuries from activities such as lifting heavy objects or, as in this case, grabbing a dog’s collar. Approximately 75% of these injuries involve the ring finger [2,3].

We report an atypical case of FDP avulsion resulting from a rare mechanism, where a large extra-articular fragment of the distal phalanx was avulsed while the patient grabbed a dog’s collar. The size and position of the bony fragment in this case were atypical, making this injury particularly noteworthy. This case highlights the importance of recognizing non-sport-related and atypical injury mechanisms to ensure appropriate and timely diagnosis, which is critical for optimal management. Early identification of such variations in injury patterns is essential to avoid misdiagnosis and to plan for the correct treatment strategy, including surgical intervention and post-operative rehabilitation.

## Case presentation

A 56-year-old right-hand-dominant female patient presented to the emergency department with pain, swelling, and inability to flex the left little finger after attempting to restrain her dog by its collar. The patient presented 12 hours after the initial injury, having experienced minimal pain and initially believing it was only a sprain. She denied any previous hand trauma and had no significant comorbidities.

Examination

**Figure 1:**
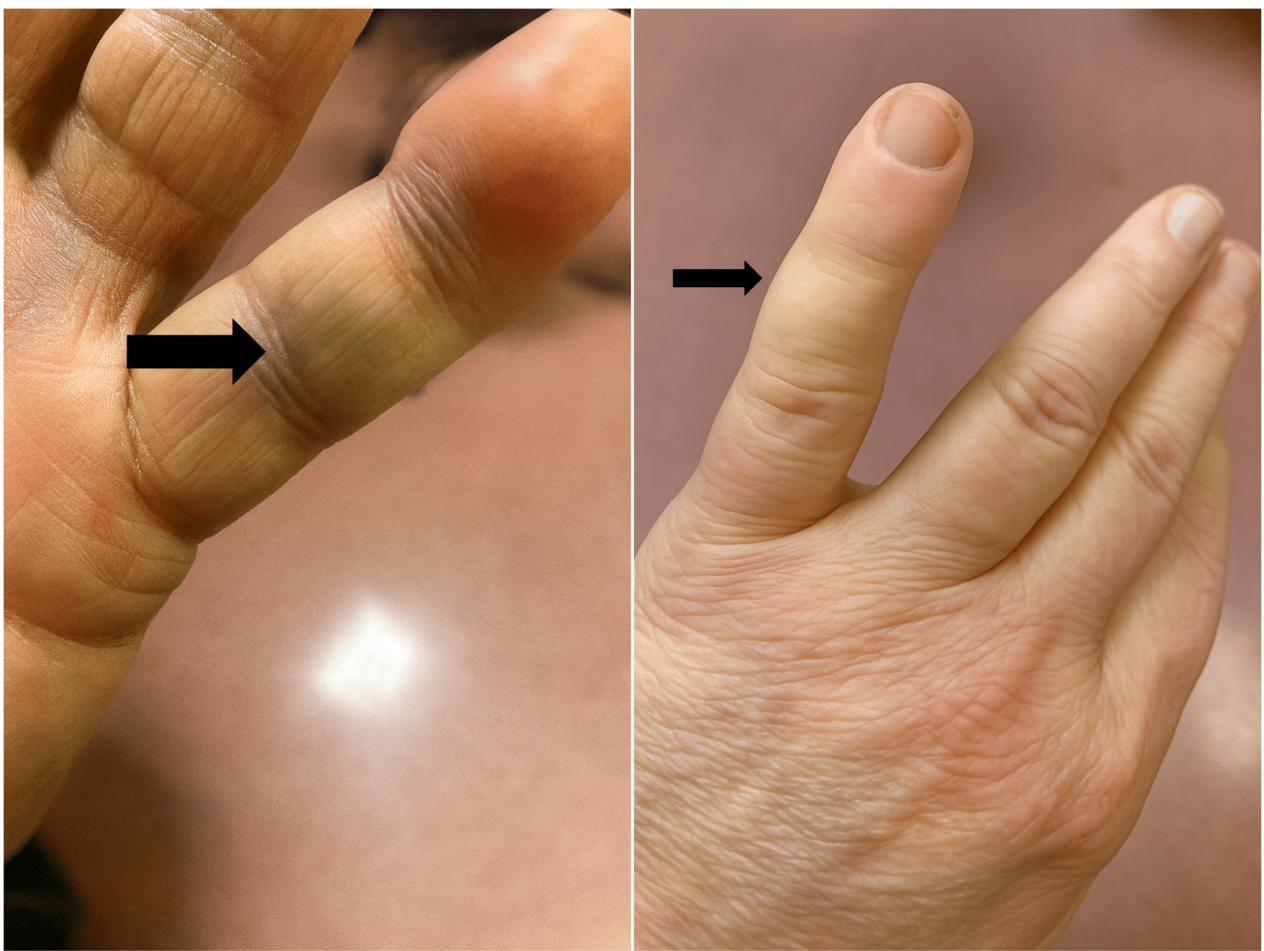
Clinical photographs of volar and dorsal aspect of the left little finger showing bruising and swelling over the middle phalanx

Swelling and bruising were noted over the middle phalanx of the left little finger (Figure 1). The patient exhibited tenderness over the volar surfaces of the middle and distal phalanges. Active flexion of the DIP joint was absent; passive flexion was preserved, albeit painful. Proximal interphalangeal (PIP) and metacarpophalangeal (MCP) joints had a full range of motion.

Imaging

**Figure 2:**
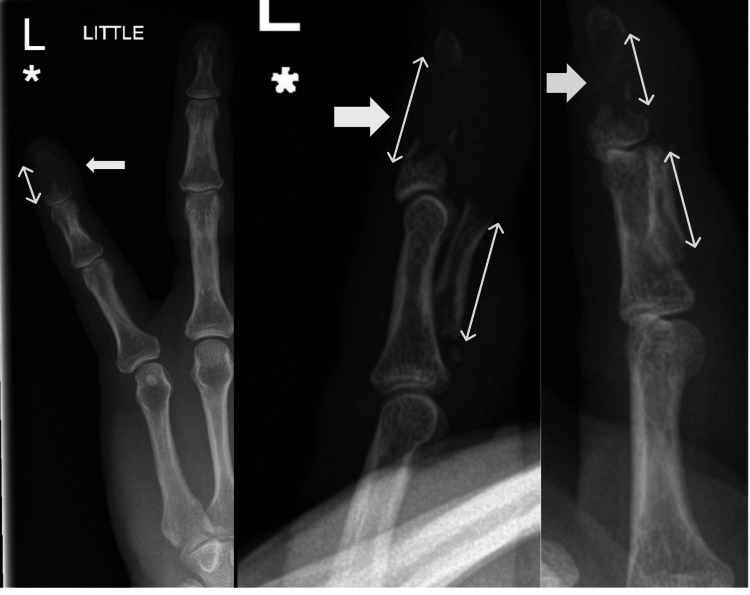
Pre-operative antero-posterior, oblique, and lateral radiographs showing large extra-articular distal phalanx fragment

Antero-posterior, oblique, and lateral radiographs revealed a large, extra-articular bony fragment from the distal phalanx, constituting approximately 80% of its length, displaced volarly and resting against the middle phalanx (Figure 2).

Management

Initially diagnosed with a distal phalangeal fracture in the accident and emergency department, the patient was subsequently found to have an FDP avulsion on further orthopaedic consultation. She was referred to a tertiary center for hand surgery and underwent surgical exploration within 72 hours. The procedure was performed using the wide-awake local anaesthetic no tourniquet (WALANT) technique, with intravenous antibiotics (as per Swansea protocol), chlorhexidine skin preparation, standard extremity draping, and adherence to the WHO surgical checklist.

**Figure 3:**
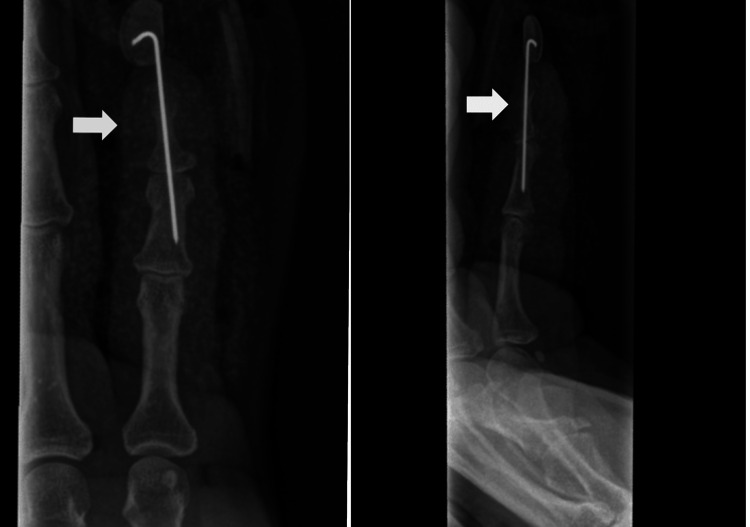
Immediate post-operative antero-posterior and lateral radiographs showing satisfactory reduction and Kirschner wire fixation

A volar Bruner incision was used to expose the proximally retracted bony fragment attached to the FDP tendon. The fragment was lodged against the middle phalanx and constrained by a partially torn A4 pulley. It was reduced to its anatomical position and stabilized using a 0.9 mm trans-articular Kirschner wire, inserted retrograde from the fingertip percutaneously via a stab incision under fluoroscopic guidance (Figure 3). No additional repair or stabilization of the FDP tendon was required. The A4 pulley was repaired.

**Figure 4:**
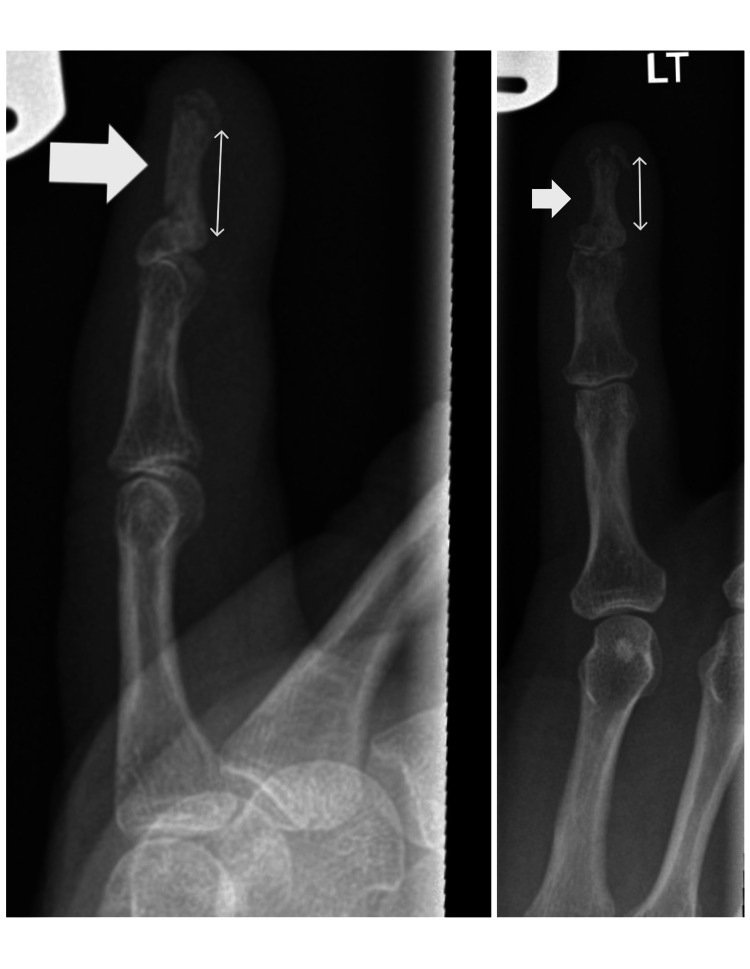
Follow-up antero-posterior and lateral radiographs at six weeks demonstrating bony union

Post-operative immobilization in a thermoplastic splint was maintained for six weeks. Supervised hand therapy was provided biweekly, following an early active range-of-motion protocol allowing mobilization of the PIP and MCP joints. At six weeks, the Kirschner wire was removed, and radiographs confirmed bony union (Figure 4). Unrestricted active extension and DIP flexion exercises were subsequently initiated.

**Figure 5:**
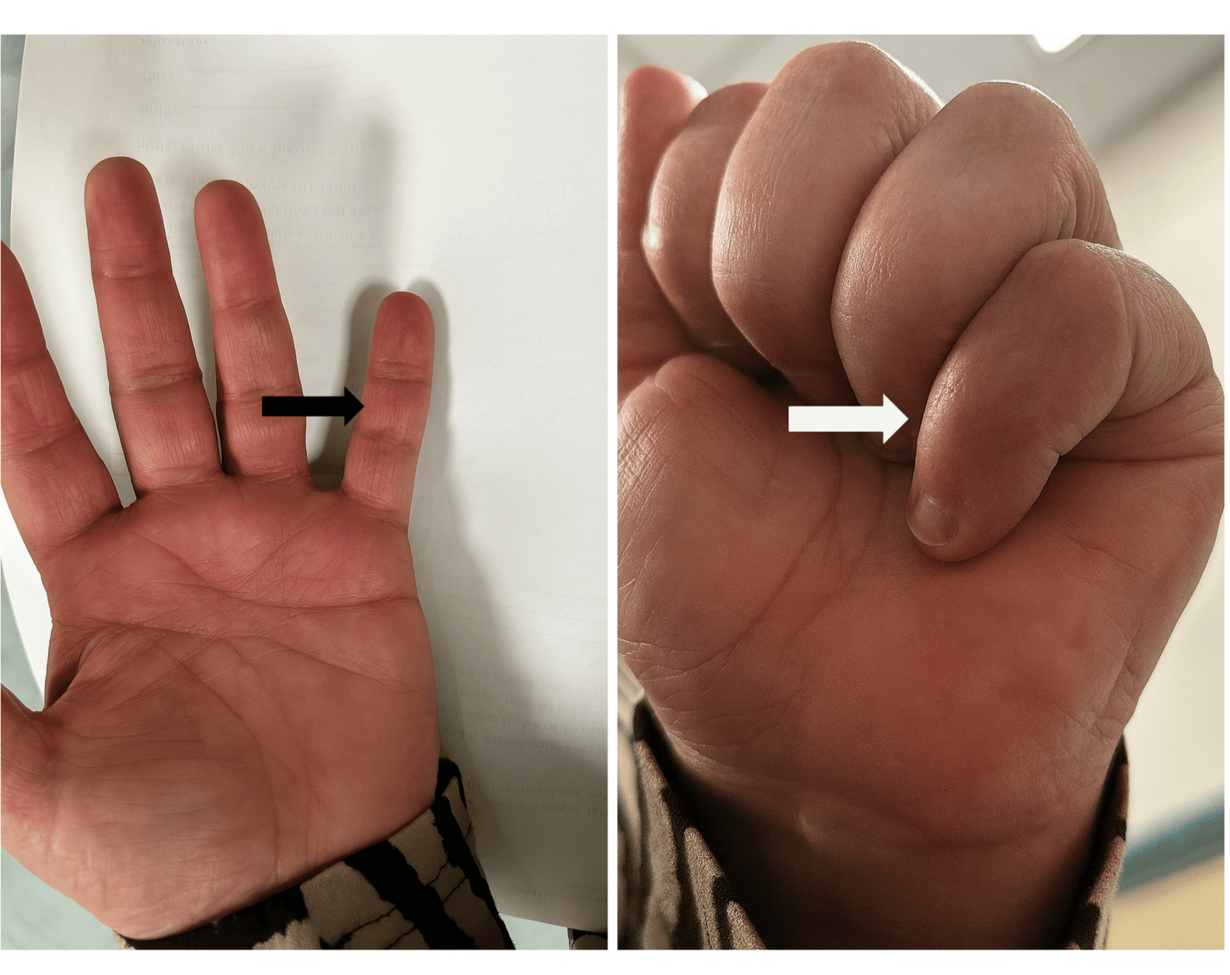
Follow-up clinical photographs showing a well-healed scar and range of motion at three months

At three months, the patient demonstrated pain-free near-full PIP range of motion and a 30° deficit in DIP flexion compared to the contralateral side. She resumed daily activities and returned to work without limitation (Figure 5). At this stage, she was discharged with hand therapy advice.

## Discussion

FDP avulsion injuries usually result from forced DIP extension during active flexion, typically seen in contact sports [2]. Although alternative mechanisms have been reported [4], this case is the first, to our knowledge, to describe injury from interaction with a dog collar. Leddy and Packer's classification (1977) remains the cornerstone in categorizing FDP avulsion injuries (Table 1) [2].

**Table 1:** Types of FDP avulsion injuries FDP: flexor digitorum profundus; PIP: proximal interphalangeal; DIP: distal interphalangeal

Types	Description
Type I	Tendon retracted to the palm without a bony fragment
Type II	Retraction to the PIP joint
Type III	Bony fragment halts retraction at the DIP level
Subsequent additions include:	
Type IV	Combined bony and tendon avulsion [4]
Type V	Large bony avulsion of the distal phalanx
Va:	Extra-articular
Vb:	Intra-articular [5]

Early surgical management is indicated in acute cases of FDP avulsion to preserve DIP flexion and hand function. Surgical technique is largely dependent on the size of the avulsed fragment and whether the tendon is still attached to the fragment or not, and these can range from a simple pull-out suture to open reduction internal fixation with tendon fixation [2,5]. Our case demonstrates an atypical Type Va-like pattern, involving a very large extra-articular fragment involving almost the whole length of the diaphysis of the distal phalanx, that was arrested by a partially ruptured A4 pulley, a detail not typically addressed in classification schemes. Previous literature has documented unusual FDP avulsions but not of this magnitude or mechanism [6-8]. In our case, early recognition allowed for prompt surgical exploration and stabilization with simple, inexpensive Kirschner wire fixation without needing any tendon fixation.

Timely recognition enabled surgical repair and preserved hand function, underscoring the necessity for high clinical suspicion in the emergency setting, especially when the mechanism and imaging are not classical. Multiple studies underscore the vital role of early imaging and clinical suspicion in facilitating an accurate diagnosis and enabling timely surgical intervention to optimize functional preservation [9].

## Conclusions

This case highlights a rare mechanism of FDP avulsion and an atypical injury pattern that deviates from established classifications. It underscores the importance of recognizing unusual anatomical variations or rare injury mechanisms that may not align with classical clinical or imaging presentations. Several studies emphasize the critical role of early imaging and clinical suspicion in ensuring accurate diagnosis and timely surgical intervention to preserve function. Maintaining a high index of suspicion, even for non-sport-related injuries, is essential. Prompt diagnosis and surgical management, including anatomical reduction and Kirschner wire fixation, can result in excellent functional outcomes.
